# Microarray analysis of gene expression in West Nile virus–infected human retinal pigment epithelium

**Published:** 2012-03-27

**Authors:** Luis Munoz-Erazo, Ricardo Natoli, Jan Marie Provis, Michelle Catherine Madigan, Nicholas Jonathan Cole King

**Affiliations:** 1 Discipline of Pathology, Bosch Institute, School of Medical Sciences, Sydney Medical School, University of Sydney, NSW, Australia; 2 Visual Sciences Group, ARC Centre of Excellence in Vision Science, Research School of Biological Sciences, NSW, Canberra, Australia; 3 Department of Anatomy, Australian National University Medical School, Canberra, Australia; 4 Optometry & Vision Science, University of New South Wales, NSW, Sydney, Australia; 5 Save Sight Institute, Sydney Eye Hospital, University of Sydney, NSW, Sydney, Australia

## Abstract

**Purpose:**

To identify key genes differentially expressed in the human retinal pigment epithelium (hRPE) following low-level West Nile virus (WNV) infection.

**Methods:**

Primary hRPE and retinal pigment epithelium cell line (ARPE-19) cells were infected with WNV (multiplicity of infection 1). RNA extracted from mock-infected and WNV-infected cells was assessed for differential expression of genes using Affymetrix microarray. Quantitative real-time PCR analysis of 23 genes was used to validate the microarray results.

**Results:**

Functional annotation clustering of the microarray data showed that gene clusters involved in immune and antiviral responses ranked highly, involving genes such as chemokine (C-C motif) ligand 2 (*CCL2*), chemokine (C-C motif) ligand 5 (*CCL5*), chemokine (C-X-C motif) ligand 10 (*CXCL10*), and toll like receptor 3 (*TLR3*). In conjunction with the quantitative real-time PCR analysis, other novel genes regulated by WNV infection included indoleamine 2,3-dioxygenase (*IDO1*), genes involved in the transforming growth factor–β pathway (bone morphogenetic protein and activin membrane-bound inhibitor homolog [*BAMBI*] and activating transcription factor 3 [*ATF3*]), and genes involved in apoptosis (tumor necrosis factor receptor superfamily, member 10d [*TNFRSF10D*]). WNV-infected RPE did not produce any interferon-γ, suggesting that *IDO1* is induced by other soluble factors, by the virus alone, or both.

**Conclusions:**

Low-level WNV infection of hRPE cells induced expression of genes that are typically associated with the host cell response to virus infection. We also identified other genes, including *IDO1* and *BAMBI,* that may influence the RPE and therefore outer blood-retinal barrier integrity during ocular infection and inflammation, or are associated with degeneration, as seen for example in aging.

## Introduction

West Nile virus (WNV) is a neurotropic flavivirus that emerged as an important human pathogen following a novel outbreak in New York City in 1999 [1]. Since then, it has spread throughout United States, Canada, and Mexico. Approximately 20% of infected persons present with a febrile illness [2] that is less commonly associated with development of encephalitis or meningitis (~1:150 infections) [3]. Ocular involvement—presenting as chorioretinitis, uveitis, occlusive retinal vasculitis, or optic neuritis [4,5]—occurs in some cases of WNV infection, although the pathogenesis and sequelae of these complications are unclear.

Viral chorioretinitis is reported to be associated with increased permeability of the retinal vasculature, degeneration of the retinal pigment epithelium (RPE), breakdown of the outer blood-retinal barrier (BRB), and migration of choroidal leukocytes into the retina [6]. To date, only one study has investigated responses of human RPE to WNV infection, showing in vitro induction of interferon (IFN)-β signaling [7].

The RPE plays a critical role in the homeostasis of the retinal environment, and in the regulation of immune responses in the choroid and retina [8-11]. It expresses a range of cell surface molecules involved in adaptive and innate immunity, as well as crucial sentinel molecules, including toll-like receptors (TLRs), which are pivotal in early cellular defense against virus infection. Furthermore, the RPE synthesizes a range of chemokines and cytokines that regulate leukocyte recruitment [12]. It is not clear whether acute responses to virus infection trigger continued proinflammatory degeneration and chronic retinal disease; some studies suggest that infection with the obligate intracellular bacterium, *Chlamydia pneumoniae* is a risk factor for age-related macular degeneration (AMD) [13,14], although the findings are somewhat contentious [15-17].

To explore the potential functional pathways that may influence RPE integrity in ocular infection and inflammation, as well as those associated with sequelae of retinal degeneration, we investigated their differential gene expression in human RPE following exposure to WNV.

## Methods

WNV (Sarafend strain, lineage II) was generated as previously described [18]. Briefly, homogenized brain supernatant from neonatal mice infected intracranially with WNV was used to infect Vero cell monolayers. Cells were then incubated for 40 h before being frozen. After thawing and centrifugation, the virus-containing supernatant was stored in aliquots at −80 °C. Virus titer was quantified in plaque-forming units, as previously described [19]. Briefly, successive dilutions of supernatant were inoculated onto baby hamster kidney–26 cell line monolayers, which were then covered with a solution of 1.5% (w/v) agarose II (Amresco, Solon, OH) and modified essential media (Sigma Aldrich, St. Louis, MO) containing 5% fetal bovine serum (FBS) for 3 days. Plaques in the cell monolayer, denoting foci of virus-induced cell death, were counted after cells were fixed with 10% formalin (Lomb Scientific, Taren Point, NSW, Australia) and stained with crystal violet 3% (Sigma Aldrich) in 20% (v/v) methanol.

Primary human RPE (hRPE) cells were cultured from postmortem eyes (n=4) obtained with consent from the Lions NSW Eye Bank (Sydney, NSW, Australia) and with ethical approval from the University of Sydney Human Research Ethics Committee, as previously described [20]. The hRPE and ARPE-19 cell line (ATCC CRL-2302; American Tissue Culture Collection, Manassas, VA) were cultured in Dulbecco’s modified essential media (DMEM; Sigma Aldrich) containing 10% FBS (Hyclone; Thermo Fischer Scientific, Waltham, MA), penicillin G (0.06 g/l; Sigma Aldrich), streptomycin sulfate (0.069 μM; Sigma Aldrich), sodium bicarbonate (0.044 M; Sigma Aldrich), and HEPES (0.02 M; Amresco) at 37 °C and with 5% CO_2_. Cells were not used beyond passage 5.

Initially, cells were seeded in six-well plates (Corning, New York, NY) at a density of 2.5×10^5^ cells/well, with eight wells seeded per donor or cell line, and allowed to attach. At 24 h post seeding, the cells were washed with PBS and six of the eight wells were infected for 1 h with WNV at a multiplicity of infection (MOI) of 0.1, 1, and 10 plaque-forming units/cell, in duplicate wells. Two wells were mock infected with DMEM only. After infection, the cells were rinsed three times with PBS and then supplemented with fresh DMEM.

For quantification of infectivity, at 24 h post WNV infection, mock-infected and WNV-infected hRPE and ARPE-19 cells were detached with 0.5% trypsin/EDTA (Sigma Aldrich). Cells were counted and 5×10^4^ cells/well were added to a round-bottomed 96-well plate (Corning). Cells were processed using the Cytofix/Cytoperm Fixation/Permeabilization Solution Kit (BD Biosciences, Franklin Lakes, NJ), and immunolabelled with either mouse anti-WNV nonstructural protein 1–fluorescein isothiocyanate (FITC)-conjugated antibody or isotype mouse IgG1 control FITC-conjugated antibody (BD Biosciences). The cells were then rinsed in FACS buffer (10% EDTA [ACR Chemical Reagent, Moorooka QLD, Australia] and 5% FBS in PBS) and filtered through Nytex (BD Biosciences) into 5 ml polystyrene tubes (BD Biosciences). The cells were processed with a BD FACSCalibur flow cytometer (BD Biosciences), and the data analyzed using FlowJo software (Version 8.8.4.; Treestar Inc., Ashland, OR).

For quantitative real-time PCR (qPCR) and microarray work, hRPE and ARPE-19 cells were infected with an MOI of 1, as above. At 24 h post WNV infection, mock-infected and WNV-infected hRPE and ARPE-19 cells were lysed with Trizol (Invitrogen). RNA was purified from the Trizol/cell lysate mixture using an RNeasy kit, as per the manufacturer’s instructions (Qiagen, Venlo, Netherlands). An additional DNase step was performed using components of the RNAqueous-Micro Kit to remove potential genomic DNA contamination (Ambion; Applied Biosciences, Melbourne, Australia). RNA concentration, contamination, and integrity (RNA integrity number value [21]) were measured using a Nanodrop ND-1000 (ThermoScientific, Waltham, MA) and a Bioanalyzer (Agilent Technologies, Santa Clara, CA). RNA extracted from the hRPE and ARPE-19 cells was of very high purity and quality (Table 1), and only samples with RNA integrity number values >9 and a 260/280 ratio >1.8 were used.

**Table 1:** RNA Integrity Numbers (RIN) and 260/280nm values for RNA samples.

**Specimen**	**RIN^a^**	**260/280 nm^b^**
hRPE1C	9.7	1.99
hRPE1V	9.6	1.98
hRPE2C	10	1.99
hRPE2V	9.4	2.05
hRPE3C	10	1.99
hRPE3V	10	2
hRPE4C	9.6	2
hRPE4V	9.6	2
ARPE-19C	9.4	2
ARPE-19V	9.6	2

RIN numbers were generated by running the RNA sample through the Aglient 2100 Bioanalyzer (Agilent). 260/280 ratios were determined by assaying the RNA samples with the Nanodrop ND1000 (Thermo Scientific). ^a^RIN - a measure of the integrity of the RNA; RIN values >9 were used. ^b^260/280 nm ratio-a measure of protein contamination; hRPE- primary human RPE; C-control, mock-infected cells; V-WNV-infected cells.

### Microarray hybridization and analysis

Target sample preparation, microarray hybridization, and scanning were performed at the ACRF Biomolecular Resource Facility in the John Curtin School of Medical Research (Australian National University, Canberra, Australia). Five control samples and five infected RPE samples were hybridized to a total of 10 Human Gene 1.0 ST chips. Processing was done using the automated GeneChip® Fluidics Station (Affymetrix, Santa Clara, CA) and scanned with the GeneChip® Scanner 3000 (Affymetrix) using an excitation laser (570 nm wavelength). CEL files were imported into the GeneSpring GX10 program (Agilent Technologies) using the robust multichip average algorithm. Preprocessing of the data was performed using the Guided Workflow feature of the GeneSpring GX10 program, which performed background correction and normalization of the probe intensities by filtering out probe intensities not within the 20th and 100th percentile. Consistency of the donor sets was established by comparing principal component analysis scores. For comparison of mock-infected and WNV-infected cells, the paired Student *t* test was used to calculate the probability (p) that the expression of a gene had not changed. Genes whose expression were significantly changed by WNV infection were selected using the criteria that p<0.05. A p value correction was not used.

Hierarchical clustering was used to study the degree of similarity or difference between the samples in this study, and was performed on the whole gene list using the GeneSpring GX10 clustering function, using the Pearson’s centered algorithm for difference measurement between conditions and Ward’s method as the criterion for linkage. Functional relationships between the genes were analyzed using the Pathway analysis feature in the GeneSpring GX10 program. Clustering of genes into functional roles or biologic processes was achieved via the functional annotation tool in the Database for Annotation, Visualization and Integrated Discovery set at medium classification stringency with a multiple linkage threshold of 0.5. Identification of biologic pathways involved in the microarray results was achieved using the pathway output function of the annotation clustering tool and Kyoto Encyclopedia of Genes and Genomes website.

### Quantitative real-time polymerase chain reaction

RNA extracted for the microarray experiments was used to generate cDNA for qPCR using SuperScript III Reverse Transcriptase (Invitrogen, Carlsbad, CA) as per the manufacturer’s instructions. Expression levels of genes of interest were determined using Taqman probes (Applied Biosystems; Table 2), Taqman Universal PCR Master mix (Applied Biosystems) as per the manufacturer’s instructions, and a StepOne Plus Real-Time PCR System with StepOne Software v2.1 (Applied Biosystems). Assays were performed in duplicate (to account for individual sample variability). Fold changes were determined using comparative cycle threshold and Delta-Delta cycle threshold. Glyceraldehyde 3-phosphate dehydrogenase (*GAPDH)* was used as a reference gene in the qPCR experiments. Amplification specificity was assessed using gel electrophoresis (data not shown). Statistical analysis was performed with REST 2009 software (Qiagen), which uses a pairwise fixed reallocation randomization test to determine significance.

**Table 2:** List of Taqman probes used in microarray validation, including genes of interest.

**Entrez Gene ID**	**Gene**	**TaqMan Probe ID**	**Amplicon length (bp)**
629	*CFB*	Hs00156060_m1	74
3075	*CFH*	Hs00164830_m1	60
7098	*TLR3*	Hs01551078_m1	132
6352	*CCL5*	Hs00174575_m1	63
6347	*CCL2*	Hs00234140_m1	101
6364	*CCL20*	Hs01011368_m1	96
3627	*CXCL10*	Hs00171042_m1	98
3569	*IL-6*	Hs00985639_m1	66
3576	*IL-8*	Hs99999034_m1	81
3456	*IFNB1*	Hs00277188_s1	134
3458	*IFNG*	Hs99999041_m1	117
7124	*TNF*	Hs00174128_m1	80
3620	*IDO*	Hs00984151_m1	68
4843	*NOS2*	Hs01075529_m1	67
8743	*TNFSF10*	Hs00921976_m1	116
8793	*TNFRSF10D*	Hs00388742_m1	93
7040	*TGFB1*	Hs00998133_m1	57
7042	*TGFB2*	Hs00234244_m1	92
25805	*BAMBI*	Hs00180818_m1	99
467	*ATF3*	Hs00231069_m1	108
3397	*ID1*	Hs00357821_g1	62
1649	*DDIT3*	Hs99999172_m1	90
2597	*GAPDH*	Hs02758991_g1	93

Taqman probes were normalized relative to the reference gene *GAPDH*, also measured with a Taqman probe. Abbreviations: CFB: complement factor B; CFH: complement factor H; TLR3: Toll-like receptor 3; CCL5: chemokine (C-C motif) ligand 5; CCL2: chemokine (C-C motif) ligand 2; CCL20: chemokine (C-C motif) ligand 20; CXCL10: chemokine (C-X-C motif) ligand 10; IL-6: interleukin 6; IL-8: interleukin-8; IFNB1: Interferon beta 1, fibroblast; IFNG: interferon gamma; TNF: tumor necrosis factor alpha; IDO: indoleamine 2,3-dioxygenase; NOS2: inducible nitric oxide synthase; TNFSF10/TRAIL: tumor necrosis factor (ligand) superfamily, member 10; TNFRSF10D: tumor necrosis factor receptor superfamily, member 10d; TGFB1: Transforming growth factor beta 1; TGFB2: Transforming growth factor beta 2; BAMBI: BMP and activin membrane-bound inhibitor homolog; ATF3: activating transcription factor 3; ID1: inhibitor of DNA binding; DDIT3: DNA-damage-inducible transcript 3; GAPDH: Glyceraldehyde 3-phosphate dehydrogenase.

## Results

**Figure 1:**
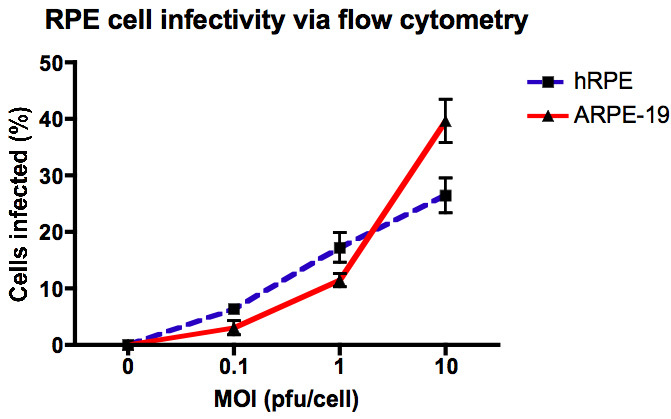
Quantification of West Nile virus infection in ARPE-19 and human retinal pigment epithelial (hRPE) cells using flow cytometry. Cells were infected with West Nile virus (WNV) at a multiplicity of infection (MOI) of 0.1, 1, and 10 (n=4), and after 24 h post infection, were fixed and permeabilized with the Cytofix/Cytoperm Fixation/Permeabilization Solution Kit (BD Biosciences) and stained with fluorescein isothiocyanate (FITC)-conjugated antibodies specific for WNV nonstructural protein 1. Cells were analyzed on a BD FACSCalibur flow cytometer. Data were analyzed using FlowJo (TreeStar software).

Flow cytometry showed that inoculation with WNV at an MOI of 1 for 24 h consistently infected approximately 20% of hRPE and 10% of ARPE-19 cells (Figure 1).

The microarray data in this publication have been uploaded to the NCBI Gene Expression Omnibus [22] and are accessible through GEO series accession number GSE30719.

### Hierarchical clustering

**Figure 2:**
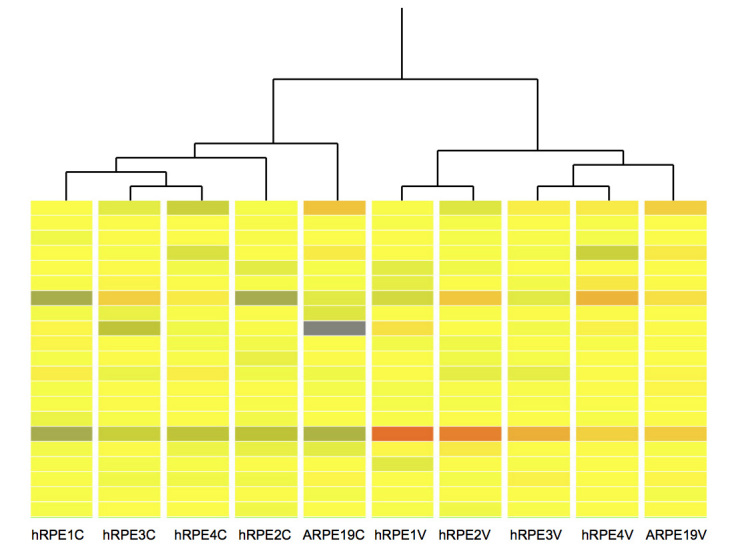
Hierarchical cluster analysis of microarray results. The columns labeled hRPE1C to hRPE4C and hRPE1V to hRPE4V are data from uninfected and West Nile virus (WNV)-infected primary human retinal pigment epithelium, respectively. ARPE-19C and ARPE-19V represent mock-infected and WNV-infected ARPE-19 cells, respectively. The data were generated using the GeneSpring GX10s (Agilent) Hierarchical Clustering feature, using the Pearson’s centered algorithm for difference measurement between conditions and Ward’s method as the criterion for linkage. ARPE-19 cells are most dissimilar from all primary hRPE cells for the mock-infected group. In the WNV-infected group, ARPE-19 cells are more similar to hRPE3 and hRPE4 cells. The image presented is an arbitrarily selected segment of the whole genome signal expression and is shown to highlight the similarity between the genome profiles between patient samples and the ARPE-19 cell line. Although not shown, there were many areas of equal similarity. Red represents high expression, yellow represents moderate expression, and blue represents low expression.

WNV-infected and mock-infected hRPE cell cultures clustered independently in two separate primary branches of the dendrogram, indicating a clear distinction in gene expression patterns for the two groups (Figure 2). The ARPE-19 cells clustered appropriately in the infected and control branches of the dendrogram. In addition, the data showed that gene expression patterns in ARPE-19 cells differ from the primary hRPE cells, which form an independent cluster (Figure 2, left branches). Despite this, infected ARPE-19 cells showed patterns of gene expression in response to infection that were similar to those seen in primary hRPE cells (Figure 2, right branches).

### Global analysis of gene expression

Differential gene expression analysis revealed 238 differentially regulated genes (fold change≥2; p<0.05 (Appendix 1). Of these, only two genes (*TNFRSF10D* and *NT5DC2*) were downregulated, all other genes being upregulated.

We performed functional clustering of these 238 genes, using the functional annotation clustering tool in the Database for Annotation, Visualization and Integrated Discovery. We selected clusters that ranked highly in proinflammatory, antiviral, apoptotic, and leukocyte recruitment processes for further investigation, because of their relevance to viral infection and retinal pathology. An abbreviated list of the genes in each of these clusters is shown in Appendix 2. The highest-ranking immune cluster (enrichment score 5.17) included: *immune response* (GO: 0050778), *immune system process* (GO: 0002684), and *response to stimulus* (GO: 0048584). The “NF-κB pathway” cluster was highly ranked, with an enrichment score of 3.57, and included: *regulation of I-κB kinase* (GO: 0043122), *protein kinase cascade* (GO: 0010740), *positive regulation of I-κB* (GO: 0043123), and *regulation of signal transduction* (GO: 0009967). Also highly ranked (enrichment score 3.37) was a “cell death cluster” that included *apoptosis* (GO: 0006915) and *regulation of cell death* (GO: 0010941). We also selected the “inflammatory cluster” (enrichment score 3.21), which included *defense response* (GO: 0006952), *inflammatory response* (GO: 0006954), and *response to wounding* (GO: 0009611).

To confirm the expression levels detected in the microarrays, using the same batch of RNA, we performed qPCR on selected genes from the above clusters using *GAPDH* as a reference gene (Table 2). Genes of interest were selected based on their representation in the ranked clusters and their likely importance to viral and retinal disease. Several of the genes of interest that we selected were represented in more than one cluster, while others were more restricted. For example, *IDO1*, *IFNB1*, and *IL-6* were represented in all clusters, *TLR3* in the *immune*, *NF-κB*, and *inflammatory* clusters, *DDIT3* and *TNFSF10* in the *NF-κB*, *inflammatory*, and *apoptosis* clusters, *CCL5*, *C3AR1*, and *CFB* in both the *immune* and *NF-κB* clusters, *TNFRSF10D* in the *inflammatory* and *apoptosis* clusters, and *CXCL10* in only the *inflammatory* cluster. We also included *CCL2* and *CCL20* in the qPCR evaluation because of their role in monocyte/macrophage/dendritic cell (DC) recruitment in WNV infection [19,23,24], even though they appeared unmodulated in the microarray data. *TGF-β1* and *TGF-β2*, as well as the associated TGF-β signaling modulator, *BAMBI*, and the TGF-β-associated transcription factor, *ATF3*, were included because of their involvement in anti-inflammatory/healing processes following acute damage, and several chronic retinal pathologies, such as proliferative retinopathy.

WNV infection of ARPE-19 cells induced patterns of gene regulation that were similar to WNV-infected hRPE cells, except that two genes (*CFB* and *BAMBI*) were more highly upregulated in hRPE compared with ARPE-19 (Table 3). Another gene—*TNFRSF10D*—was upregulated in infected ARPE-19 cells, but downregulated in hRPE cells. All other genes analyzed were modulated in similar directions in both hRPE and ARPE-19 cells.

**Table 3:** Fold changes in selected gene expression determined by qPCR for WNV-infected primary hRPE versus ARPE-19 cells.

**Gene**	**ARPE19**	**hRPE1**	**hRPE2**	**hRPE3**	**hRPE4**
*TNFRS10D*	5.404	0.16	0.16	0.292	0.209
*CFB*	3.334	16.225	15.199	6.038	39.097
*BAMBI*	2.316	290.042	13.641	10.797	187.345

Genes with the most dissimilar fold changes between ARPE-19 and the primary retinal pigment epithelium (RPE) cell groups are shown. *TNFRS10D* is the most dissimilar, being upregulated in ARPE-19 cells, compared to all other primary RPE groups, which were downregulated. Both *CFB* and *BAMBI* showed differences only in the magnitude of upregulation between ARPE-19 and primary RPE groups. Abbreviations: TNFRSF10D: tumor necrosis factor receptor superfamily, member 10d; CFB: complement factor B; BAMBI: BMP and activin membrane-bound inhibitor homolog.

**Figure 3:**
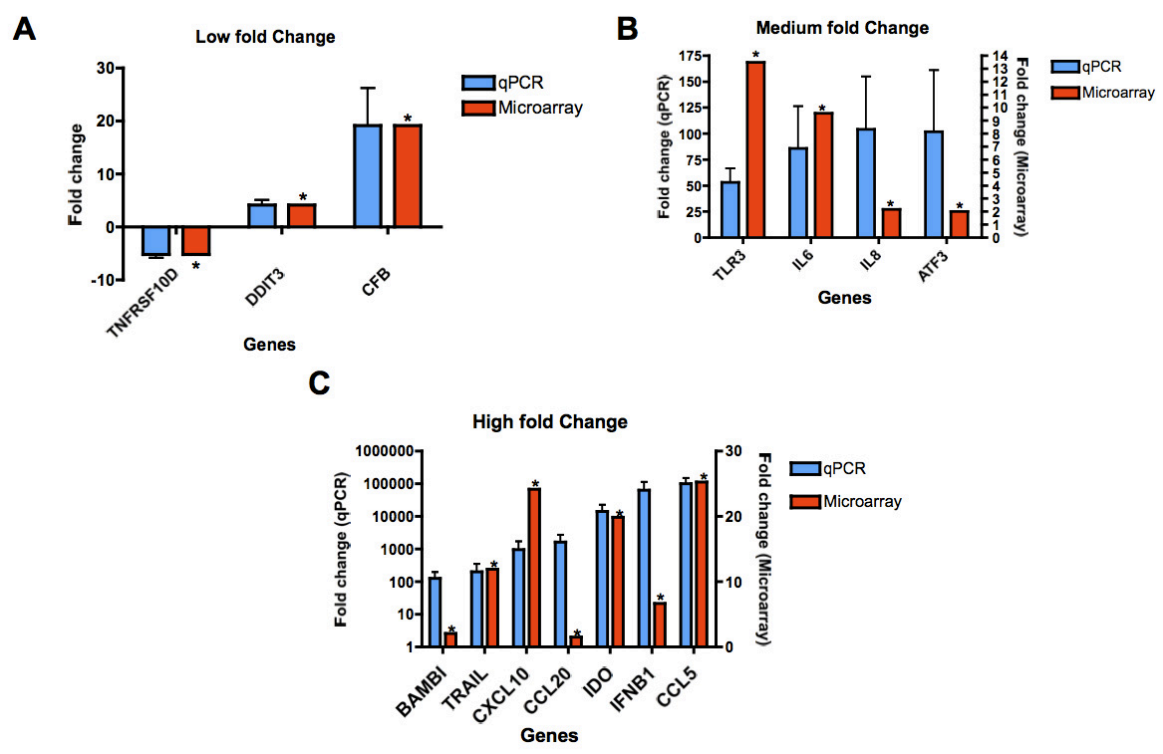
Comparison of differential gene expression with microarray (red) and qPCR (blue). Genes are allocated to the graphs shown here, according to fold-change of expression after WNV infection, as determined by qPCR, with microarray values shown adjacent to these. **A**: qPCR fold change −10 to 20; (B) qPCR fold change 20 to 120, and (C) qPCR fold change >120. qPCR reactions were performed in duplicate and both qPCR and microarray data used the same RNA as the source. Samples were isolated from 4 separate donors. Genes in qPCR were amplified using Taqman probes and Taqman Universal PCR master mix (Applied Biosystems). *p<0.05 as evaluated by paired Student *t*-test, compared to matched, uninfected donor samples.

Figure 3A-C compares gene regulation in hRPE cells assessed by microarray (red) and qPCR (blue). While highly upregulated genes varied in their fold changes between microarray and qPCR results (*CCL5*, *IDO1* and *TLR3*), there was a clear consistency in directional change, with good correlation between the two techniques for small changes in gene expression.

**Figure 4:**
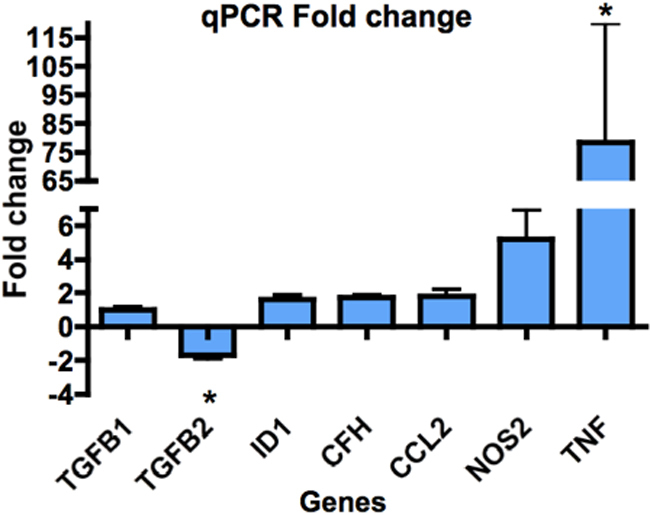
Fold changes in expression of genes not seen to change on the microarray, as shown by qPCR. All qPCR reactions were performed in duplicate on cDNA extracted from 4 separate donors. Genes were amplified using Taqman probes and Taqman Universal PCR master mix (Applied Biosystems). Genes were selected for further qPCR analysis, based on the immune and TGF-β gene groups of interest seen in the microarray analysis. Statistical analysis was undertaken using the REST program (Qiagen), which uses a pair-wise fixed reallocation randomization test to determine significance. Averaged values of duplicate samples from 4 separate donors were statistically analyzed. However, despite appreciable fold-changes in some samples, only *TNF* and *TGF-β2* genes in WNV-infected RPE were statistically different from uninfected RPE. * represents p<0.05.

Figure 4 shows the genes that were not altered in the microarray output but were when analyzed by qPCR. Despite appreciable fold changes in some samples, on analysis only the *TNF* and *TGFB2* genes in the WNV-infected RPE were statistically different from the uninfected RPE.

The gene showing the largest downregulation was tumor necrosis factor (TNF) receptor superfamily member 10D (*TNFRSF10D*, *TRAILR4*), which showed a consistent downregulation of 2.39-fold in the microarray and 4.76-fold in the qPCR results (Figure 3A). For all genes with >20-fold expression changes, qPCR showed greater changes than in the microarray (Figure 3C). The chemokine *CCL5* (RANTES) showed the largest differential expression, with a 25.23-fold increase in the microarray and a >70,000-fold increase using qPCR (Figure 3C). Similarly, the immunomodulatory enzyme, indoleamine 2,3-dioxygenase 1 (*IDO1*) showed a 19-fold increase in the microarray and a >14,000-fold increase in the qPCR (Figure 3C). The gene *IFNG* was not detected by qPCR in either mock-infected or infected cells.

## Discussion

This is the first study to investigate the global transcriptome in WNV-exposed RPE. Because the RPE maintains the outer BRB, its response to WNV infection is important. WNV infection is likely to compromise the functional integrity of the RPE, permitting infection of the neural retina.

Currently, there are no definitive risk factors associated with individuals afflicted with WNV-induced ocular pathologies. A review of the WNV cases involving ocular manifestations in the current literature where individual patient ages are provided reveals that the ages of patients range from 28 to 86 years, with a mean of 57.6 and a median of 56.5 years (n=18). A study of WNV-induced ocular pathology by Khairallah et al. [25] reported a mean age of 47.2 years (n=12). A review of the patient medical histories, where available, indicates that up to 51.5% had diabetes of some sort. Given that diabetes type 1 is a type of autoimmunity [26] and there is evidence to suggest that type 2 diabetes alters the immune system [27], the pathways found in our study highlight the role of the immune system in BRB breakdown. Furthermore, several other autoimmune disorders can lead to outer BRB breakdown, including rheumatoid arthritis, sarcoidosis, and systemic lupus erythematosus [28]. Our findings show that the key differentially expressed genes are those involved in immune, NF-κB, inflammatory, and apoptotic processes consistent with innate immunological responses to virus infection. Several gene groups and key genes important in reducing local viral infection are identified, including complement-related genes *C3AR1* and *CFB*, as well as *TLR3*, *IFNB1* [29,30], genes encoding chemokines for recruiting neutrophils (*IL-8*), monocytes (*CCL2*), dendritic cells (*CCL20*), T cells and NK cells (*CCL5, CCL20, CXCL10*) [31], and genes involved in modulation of the immune response (*IDO1*) [32]. Changes in the expression profiles of genes involved in the TGF-β pathway (*BAMBI* and *ATF3*) and cell death (*TNFRS10D*) are also reported.

### Complement pathway

The complement system is involved in both direct and indirect mechanisms of pathogen clearance [33], and various complement components contribute to WNV neutralization by directly binding virus and by enhancing antibody-mediated viral clearance [34]. Interestingly, the complement cascade is implicated in the pathogenesis of AMD [35,36], indicating a role for complement in ocular inflammatory disease. We showed a significant increase in expression for *CFB* in the microarray and a nonsignificant increase in *CFH* by qPCR following WNV infection. Although *C3* showed no differential regulation on the microarray, its receptor gene, *C3AR1*, was upregulated (Appendix 1), suggesting an overall proinflammatory response following low-level WNV infection.

### Chemokines

The chemokines observed to be upregulated by WNV infection of RPE cells have roles in the recruitment of leukocyte subsets, consistent with the pathogenesis and resolution of WNV and other viral infections. These leukocyte subsets include monocytes recruited by CCL2 [19,37], T cells recruited by CCL5 [31], T cells and NK cells recruited by CXCL10 [38,39], dendritic cells recruited by CCL20 [40], and neutrophils recruited by IL-8 [41]. Some of these chemokines have also been investigated in retinal pathologies or associated with WNV infection. For example, Ambati et al. [42] found that CCL2 and CCR2 (a CCL2 receptor) knockout mice developed histopathological features typical of dry AMD, including drusen deposits, Bruch’s membrane thickening, and complement deposition, consistent with an immunoregulatory role for CCL2. Additionally, CCL2 has been reported to increase the in vitro and in vivo permeability of the blood-brain barrier [43], with implications for the BRB, as well as being responsible for the recruitment of pathogenic inflammatory monocytes to the brain in WNV encephalitis [19]. CCL5 is also a key chemokine in WNV infection [44-46]. Sonoda et al. [47] found that neutralization of macrophage-derived CCL5 exacerbated murine experimental autoimmune uveitis, most likely by altering the composition of the T cell subsets recruited. CXCL10 is reported to be upregulated in AMD patients when compared to age-matched controls, with peak expression at the intermediate stage of the disease [48].

Taken together, these observations suggest that aberrant expression of some or all of these chemokines is involved in ocular disease associated with dysregulated leukocyte recruitment, BRB breakdown, and choroidal neovascularization.

### Other cytokines

WNV infection has previously been shown to induce IFN-β1 production, which subsequently plays a role in inhibiting further WNV infection in the RPE [7]. The upregulation of *IFNB1* observed in our study supports these earlier observations. Genes such as *CXCL10* [49], *IL-8* [50], *NOS2,* and *IDO1* [51] have been shown to be upregulated by IFN-γ, which we subsequently investigated using qPCR. As expected, since IFN-γ is a type II IFN produced by lymphocytes such as T cells and NK cells [52,53], *IFN-γ* mRNA was not detected in either mock-infected or WNV-infected RPE cells, indicating that these factors are induced by other pathways.

The microarray data did not indicate regulation of *TNF* expression. However, since TNF-associated viral response genes were upregulated, we further investigated *TNF* expression using qPCR, finding it to be highly upregulated in WNV-infected RPE cells. The relevance of TNF in retinal pathologies has been seen in several studies. For example, TNF has a role in the apoptosis of RPE and photoreceptor apoptosis in murine cytomegalovirus retinitis [54], and Takahashi et al. [55] concluded that TNF has a role in RPE epithelial-mesenchymal transition (discussed below in terms of TGF-β genes).

### Genes involved in the immune response

TLR3, a receptor for dsRNA, has a major role in promoting antiviral responses [56] and is highly expressed in RPE [57]. We found a >50-fold upregulation of *TLR3* expression in RPE following WNV infection, indicating a robust antiviral response by the RPE. In the central nervous system, *TLR3* modulates the WNV-related inflammatory response and subsequent blood-brain barrier breakdown, as well as influencing central nervous system viral replication and neuronal injury [58].

An emerging gene of interest is *IDO1*, which has antimicrobial activity via cellular L-tryptophan depletion [59-61]. In RPE cells, IDO1 activity has been shown to inhibit the growth of *Toxoplasma gondii* [62] and cytomegalovirus [51]. Conversely, IDO1 is also immunoregulatory via L-tryptophan depletion and can arrest the T cell proliferation, induce regulatory T-cells, impair generation of memory T-cells [32], and prime dendritic cells to induce tolerance [63]. Impaired IDO1 activity may thus lead to inefficient viral clearance and/or uncontrolled inflammatory leukocyte activity.

NOS2 is one of the three isotypes of nitric oxide (NO)-catalyzing genes. NO has antimicrobial effects [64], and roles in signal transduction and vasodilatation [65]. NO is important for pathogen clearance, and given its cytotoxicity, sustained NO production may induce pathological effects in the RPE, including cell death and altered barrier integrity [66]. Like IDO1, NOS2 has an immunoregulatory role associated with leukocytes [67,68]. The activity of these enzymes is believed to be interlinked, with certain immune processes favoring production of one enzyme over the other [69]. Given that *IDO1* is increased to a significantly greater extent compared to *NOS2* (Figure 3C, Figure 4), it is tempting to speculate that a pro-IDO1 regulatory response in the WNV-infected RPE may better preserve RPE barrier function in vivo both by nondestructively inhibiting viral replication and inhibiting local T cell responses.

### Regulation of cell death associated with West Nile virus infection

RPE cell death may result in the functional impairment and death of retinal photoreceptors [70], as evidenced by the RPE atrophy, photoreceptor loss, and secondary degeneration of the choriocapillaris seen in atrophic AMD [71]. Given the importance of RPE integrity in retinal function, we investigated genes involved in cell death.

We found upregulation of a proapoptotic gene, *TNFSF10/TRAIL*, and decreased expression of the antiapoptotic *TNFRSF10D* receptor. This receptor has a truncated death domain that can inhibit the apoptotic effects of TNFSF10/TRAIL [72]. These observations suggest that RPE cells infected with low-level WNV may be primed for apoptosis. A similar inverse relationship between TNFSF10 and TNFRSF10D is seen in primary human fibroblast cells treated with the antiviral cytokines IFN-γ and IFN-β1 [73]. It was concluded that these changes may contribute toward apoptosis-mediated resolution of HSV-1 infection in these cells [73]. This suggests a potential role for WNV-induced cell death.

### Transforming growth factor–β genes

Genes involved in the TGF-β pathway were also identified in the microarray, in particular *BAMBI*, *ATF3*, and *ID1*. To further explore these pathways, we also investigated the expression of *TGFB1* and -*2* via qPCR. *TGFB1* did not show any change at 24 h post WNV infection; however, *TGFB2*, the predominant isoform of TGF-β in the eye [74-76], and the major isoform produced by the RPE [77], was downregulated (Figure 4).

**Figure 5:**
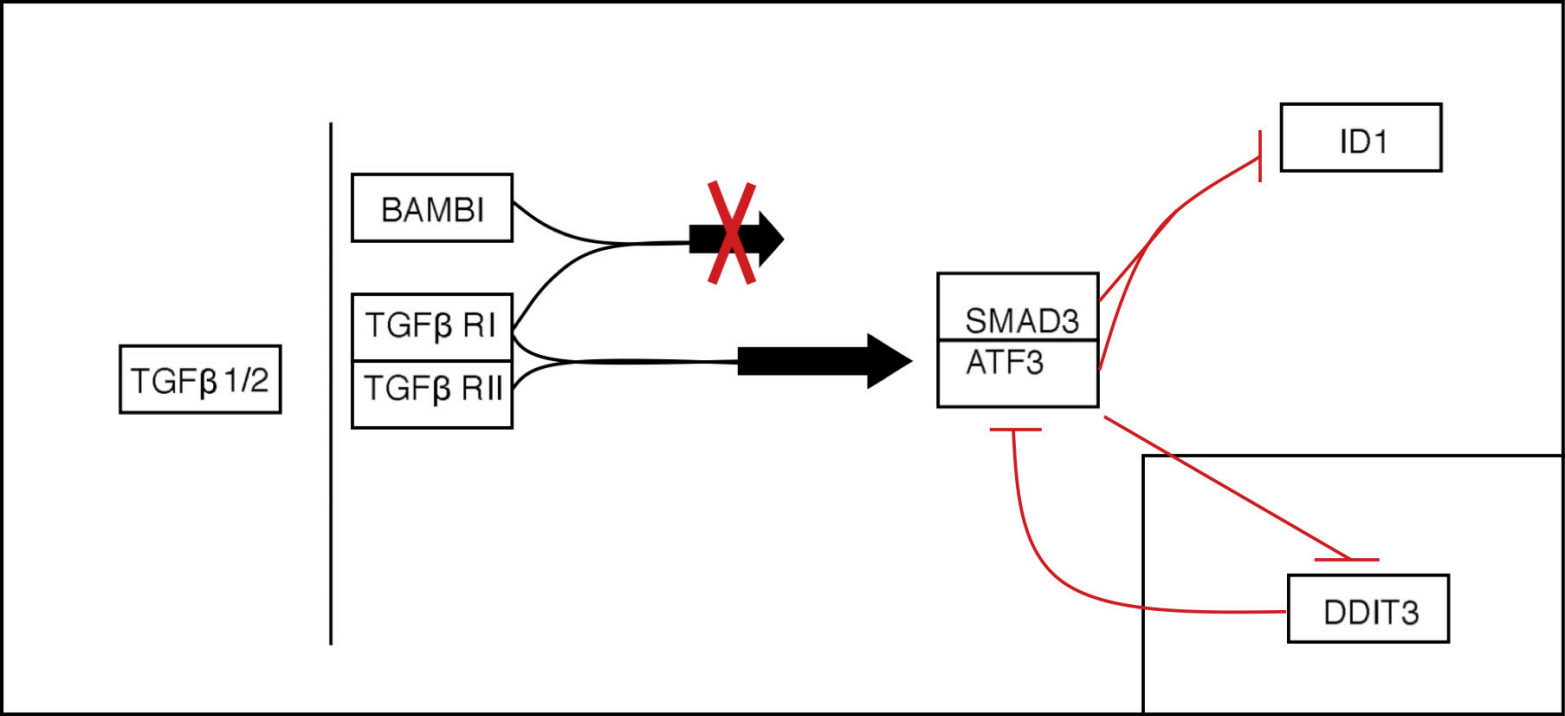
Simplified TGF-β pathway. Several genes in this pathway are included in our analysis (*TGFβ 1/2*, *BAMBI*, *ATF3*, *ID1*), as well as associated genes that were not analyzed (TGF-β RI/II, SMAD3). TGF-β1 or 2 binds to the TGF-β RI and RII receptor complex, and results in signal transduction. However, if the TGFβ ligand binds to a BAMBI/TGF-β RI complex, this signal is not transduced. TGF-β signaling can lead to the binding of SMAD3 with ATF3. This complex can go on to repress the transcription of *ID1*. Independently of the TGF-β pathway, DDIT3 protein can inhibit the transcription of *ATF3*, while ATF3 can also suppress the transcription of *DDIT3* (insert).

In the eye, the TGF-β pathways are multifunctional, with roles in angiogenesis [78] and immune regulation [79]. High levels of TGF-β can lead to pathological responses such as intraocular fibrosis and proliferative vitreoretinopathy [80]. *BAMBI* and *ATF3* are involved as a signal modulator and transcription factor, respectively, in the TGF-β signaling pathway (Figure 5). The BMP and activin membrane-bound inhibitor homolog (BAMBI) belongs to the TGF-β superfamily [81], and is a negative regulator of TGF-β family signaling [82]. The downstream effects of BAMBI are not fully understood. In vitro studies of human biliary epithelial cells showed downregulation of BAMBI in response to poly (I:C), a viral dsRNA analog, suggesting that dsRNA virus infection could lead to loss of cell-to-cell adhesion and epithelial mesenchymal transition (EMT) [83]. As cell junction disintegration is a feature of EMT [84], this could compromise the RPE, and thus BRB integrity. However, WNV infection of RPE was associated with significant upregulation of BAMBI in the first 24 h. As a host response, this may be important in limiting the pathological effects of TGF-β signaling in the retina.

ATF3 regulates a variety of pathways, including two identified in the functional analysis of our microarray data—the TGF-β signaling pathway and one involving DDIT3. In the TGF-β signaling pathway, a SMAD3/4 complex normally leads to the transcription of *ID1*, which inhibits proliferation but results in differentiation in a variety of cell types [85-87]. However, induction of ATF3 can result in repression of *ID1* [88], leading cells back to a proliferative state (Figure 5). The balance of ATF3 and ID1 may therefore play a role in determining the type of response elicited in RPE cells (e.g., proliferation, EMT, etc.,) following pathogenic insult.

### Comparisons between the primary human retinal pigment epithelium and the ARPE-19 cell line

Given the difficulty in obtaining primary hRPE cells, many laboratories use ARPE-19 cells to study the responses of RPE in various conditions. WNV infection of ARPE-19 cells in the current study generally induced similar patterns of gene expression regulation to WNV-infected hRPE cells. However, certain genes showed a difference in regulation; for example ARPE-19 cells showed upregulation of *TNFRSF10D*, in contrast to downregulation found in hRPE. Although not discussed by Leung et al., a similar differential expression is seen in the production of IL-13 by hRPE when compared to ARPE-19 [89]. Together with our results, this observation suggests that a level of caution is necessary in translating results from the ARPE-19 cell line to primary hRPE.

### Conclusions

This study identified differentially expressed genes associated with WNV infection in RPE. These findings may be useful in identifying previously unknown factors that influence outer BRB integrity and contribute to the clinical presentation of ocular disease, as well as potential downstream sequelae of ocular viral infection. This study also provides a framework for future discovery of the roles of individual genes in these processes.

## Appendix 1. Table of differentially regulated genes in WNV-infected RPE with a twofold or greater change, as determined by microarray.

To access the data, click or select the words “Appendix 1.” This will initiate the download of a compressed (pdf) archive that contains the file.

## Appendix 2. Highly represented clusters of genes in the differential expression of data.

To access the data, click or select the words “Appendix 2.” This will initiate the download of a compressed (pdf) archive that contains the file. This table is an abbreviated version of the output generated from microarray data analyzed via the functional clustering tool on the Database for Annotation, Visualization and Integrated Discovery (DAVID) website. The main biologic processes represented in the table relate to immune response and to apoptosis. The biologic process ‘response to bacterium’ appears likely due to the common genes shared by this biologic cluster and genes involved in viral response. *Abbreviation:* Gene Ontology (GO).
